# The Tyrosine Kinase Receptor ROR1 Is Constitutively Phosphorylated in Chronic Lymphocytic Leukemia (CLL) Cells

**DOI:** 10.1371/journal.pone.0078339

**Published:** 2013-10-24

**Authors:** Mohammad Hojjat-Farsangi, Abdul Salam Khan, Amir Hossein Daneshmanesh, Ali Moshfegh, Åsa Sandin, Ladan Mansouri, Marzia Palma, Jeanette Lundin, Anders Österborg, Håkan Mellstedt

**Affiliations:** 1 Department of Oncology-Pathology, Immune and Gene therapy Lab, Cancer Center Karolinska (CCK), Karolinska University Hospital Solna and Karolinska Institute, Stockholm, Sweden; 2 Departments of Oncology and Hematology, Karolinska University Hospital Solna, Stockholm, Sweden; University of Thessaly, Faculty of Medicine, Greece

## Abstract

Phosphorylation of receptor tyrosine kinases (RTKs) has a key role in cellular functions contributing to the malignant phenotype of tumor cells. We and others have previously demonstrated that RTK ROR1 is overexpressed in chronic lymphocytic leukemia (CLL). Silencing siRNA downregulated ROR1 and induced apoptosis of CLL cells. In the present study we analysed ROR1 isoforms and the phosphorylation pattern in CLL cells (n=38) applying western blot and flow-cytometry using anti-ROR1 antibodies and an anti-phospho-ROR1 antibody against the TK domain. Two major ROR1 bands with the size of 105 and 130 kDa respectively were identified, presumably representing unglycosylated (immature) and glycosylated (mature) ROR1 respectively as well as a 260 kDa band which may represent dimerized ROR1. A ROR1 band of 64 kDa that may correspond to a C-terminal fragment was also noted, present only in the nucleus. The 105 kDa ROR1 isoform was more frequently expressed in non-progressive as compared to progressive CLL patients (p=0.03). The 64, 105, 130 and 260 kDa bands were constitutively phosphorylated both at tyrosine and serine residues. Phosphorylation intensity of the mature (130 kDa) isoform was significantly higher in progressive than in non-progressive disease (p<0.001). Incubation of CLL cells with a mouse anti-ROR1 KNG or an anti-ROR1 CRD mAb respectively induced dephosphorylation of ROR1 before entering apoptosis. In conclusion CLL cells expressed different isoforms of ROR1 which were constitutively phosphorylated. The mature, phosphorylated ROR1 isoform was associated with a progressive disease stage. Targeting ROR1 by mAbs induced specific dephosphorylation and leukemic cell death. ROR1 might be an interesting therapeutic target.

## Introduction

The receptor tyrosine kinase like orphan receptor 1 (ROR1) belongs to one of the twenty receptor tyrosine kinase (RTK) families and is evolutionarily highly conserved among different species [1]. ROR1 is structurally related to other RTKs as the Trk family and the muscle-specific kinase (MuSK). It is expressed early during embryogenesis. The protein level peaks at periods of active synapse formation, concentrated in the growth cones of immature neurons and present throughout the somatodendritic part of mature hippocampal cells [2].

The human *ROR1* gene is located on chromosome 1 (1p31.3) (http://www.ensemble.org) with a coding region of 2814 bp and 937 amino acids (full length variant). The protein consists of an extracellular part including 3 domains; the immunoglobulin (Ig) like, the cysteine-rich (CRD) and the kringle (KNG) domains as well as an intracellular tyrosine kinase and a proline-rich domain. The *ROR1* gene was originally cloned from a neuroblastoma cell-line [1]. Wnt5a is a ligand for ROR2 and activates canonical and non-canonical pathways by binding to the frizzled 2 domain of ROR2 [3] and was also suggested to be a ligand for ROR1. However, other Wnt proteins may also be considered [3].

Different isoforms of ROR1 has been reported (http://www.ensembl.org) [4]. One of the isoforms is the full length core protein, 937 aa (100-105 kDa unglycosylated) (ROR1-001, ENSP00000360120). Another has a length of 393 aa (about 40 kDa) (ROR1-002, ENSP00000360121) and a third of 388 aa (ROR1-201, ENSP00000441637). A truncated ROR1 gene has been found in the fetal and adult human central nervous system, in human leukemia and lymphoma cell-lines and in a variety of human cancer derived from the neuroectoderm with a predicted protein size of 40 kDa [4]. A 50 kDa ROR1 isoform has also been reported which may originate from a splice variant [5].

We and others have demonstrated the overexpression of the ROR1 protein in chronic lymphocytic leukemia (CLL) and other lymphoid malignancies [5-7]. The expression was uniform [5,8] and higher in progressive disease [8].

Post-translational modifications of proteins are important for functional characteristics. Different ROR1 glycosylation patterns have been reported in CLL. The fully glycosylated isoform (130 kDa) has been suggested to be surface bound, but it is unclear for the unglycosylated isoform (100-105 kDa) [6,9]. Partially glycosylated isoforms have also been reported [9].

Phosphorylation of RTKs, especially of tyrosine residues, is an important mechanism for regulation of cellular functions including cell division, protein synthesis and transcriptional regulation. Phosphorylation at tyrosine or serine residues may have various functional implications [10]. One third of all cellular proteins and >95% of RTKs are phosphorylated at tyrosine, serine and/or threonine residues [11]. Although phosphorylation at tyrosine is less common (1.8%) than at serine (86.4%) or threonine (11.8%) residues, tyrosine phosphorylation is of significant importance in health and disease [12]. 

In the present study, we analysed different ROR1 isoforms and the phosphorylation status in CLL patients in various clinical phases of the disease. We also evaluated the effects of two apoptosis inducing anti-ROR1 mAbs (anti-KNG 4A7 and anti-CRD 1D8) [8] on ROR1 phosphorylation. We demonstrated that various ROR1 isoforms were differentially expressed in non-progressive vs. progressive CLL patients and ROR1 was constitutively phosphorylated particularly in progressive CLL patients. Dephosphorylation of ROR1 preceded in vitro apoptosis induced by the anti-ROR1 mAbs. 

## Materials and Methods

### Patients

Thirty eight CLL patients (21 with progressive and 17 with non-progressive disease) were included in the study. The study was approved by the Regional Ethics Committee (www.epn.se) and signed written informed consent was obtained from each patient prior to blood sampling. The diagnostic criteria for CLL have been reported earlier [13]. Definition of non-progressive (NP) and progressive (P) disease at the time of testing have been described previously [13]. Peripheral blood mononuclear cells (PBMC) and purified B-cells of healthy individuals were used as controls.

### Isolation of PBMC and B cells

PBMC were isolated using Ficoll-Hypaque (GE Healthcare, Uppsala, Sweden) density-gradient centrifugation. B-cells from healthy donors were isolated from PBMC using MACS negative selection kits (Miltenyi Biotec, Bergisch Gladbach, Germany) according to the manufacturer’s instructions as previously described [14]. CD19 positive cells were enriched by negative selection using MACS microbeads and midiMACS columns. The purity of cells was >95% as determined by flow cytometry.

### Flow cytometric surface and cytoplasmic staining

Surface staining of CLL cells was performed as described [6,15]. Briefly, 10^6^ cells were washed in PBS and suspended in 100 µl of FACS buffer (PBS with 0.1% sodium azide). A polyclonal goat anti-ROR1 antibody (Ab) (3 µg) (against the extracellular part of ROR1) (R&D system, Minneapolis, MN, USA) and a goat IgG as a non-relevant isotype control Ab (3 µg) (Santa Cruz Biotechnology, Santa Cruz, CA, USA) were added to the cells and incubated at 4 °C for 1 h before washing twice with FACS buffer. The secondary antibody, FITC rabbit anti-goat IgG (1:50) (Dako Cytomation, Glostrup, Denmark), was added and incubated at 4 °C for 1 h, washed twice in FACS buffer and fixed with 1% paraformaldehyde in PBS. Intracytoplasmic staining of leukemic and normal PBMC was performed as previously described [16]. A LSR II flow cytometer (BD Biosciences, Mountain View, CA, USA) was used and the data were analysed by FlowJo software program (Tree Star Inc. Ashland, OR, USA). 

### Apoptosis assay

Apoptosis assay was done as previously described [8]. Briefly, 10^6^ CLL cells were incubated with 10 µg/ml of two self-produced anti-ROR1 monoclonal antibodies (mAbs) (against the extracellular KNG and CRD domains of ROR1, respectively) [8] or a non-relevant IgG1 isotype control mAb (10 ug/ml) or a human anti-CD20 Ab (ofatumumab) (10 ug/ml) (GlaxoSmithKline, Research Triangle Park, NC, USA) in RPMI 1640 containing FCS. After 20 min, 1, 4, 12 and 18 h of incubation at 37 °C, 10^5^ cells were collected, washed twice in PBS and resuspended in 100 ul of binding buffer. Five µl of FITC-conjugated Annexin V and PI (BD Biosciences) was added. The tubes were vortexed and incubated at room temperature in the dark for 15 min. One hundred µl of binding buffer was added to the cells which then were analyzed by flow cytometry. Apoptotic/necrotic cells were determined as the Annexin V^+^/PI^+^ population. Cell lysates were prepared and stored at -20 °C for further analysis. 

### Immunoprecipitation of ROR1

Immunoprecipitation of ROR1 was done using a rat monoclonal anti-human ROR1 antibody (against the extracellular part of ROR1) (R&D). Briefly, 500 µg of cell lysate was precleared with a mixture of protein G and protein A agarose (Calbiochem, Darmstadt, Germany) at 4 °C for 2 h. The supernatant was collected and incubated with protein G, protein A agarose and 3 µg of the rat anti-human ROR1 mAb at 4 °C for overnight. Finally, precipitated agarose beads were washed 3 times with lysis buffer and suspended in sample buffer for further analysis by western blot. 

### Western blot analysis

PBMC from CLL patients or B-cells from healthy control donors were lysed in buffer containing 150 mM NaCl, 25 mM Tris-HCl, 1% NP-40, 1% Na deoxycholate, 0.1% sodium dodecyl sulfate (SDS), 1% phosphatase inhibitor (Roche Ltd, Basel, Switzerland) and 1% protease inhibitor cocktail (Sigma-Aldrich Corp., Saint Louis, MO). Western blot was performed as previously described [8]. Briefly, protein concentration of the lysate was measured using BCA Protein Assay Kit according to manufacturer’s instructions (Thermo Scientific, Barrington, IL, USA). Twenty µg of cell lysate or immunoprecipitated ROR1 was run on 4-10% (gradient) or 10% Bis-Tris SDS-PAGE gels (Invitrogen, Carlsbad, CA, USA) under non-reducing and reducing (for immunoprecipitated ROR1) conditions. After electrophoresis, resolved proteins were transferred to Immobilon-PVDF membranes (Millipore Corporation, Bedford, MA, USA) in a mini Transblot cell (Invitrogen). The membranes were blocked at 4 °C with 5% non-fat milk or 5% BSA in TBS containing 0.1% Tween 20 (TBS-T) overnight. Filters were incubated with an anti-pROR1 mAb (against the intracellular tyrosine kinase domain of ROR1) (a gift from Monoclonal Antibody Research Center, Avicenna Research Institute, Tehran, Iran), anti-phospho-tyrosine mAb (clone 4G10), anti-phospho-serine mAb (clone 4A4) (Millipore) and a polyclonal goat anti-human ROR1 Ab (R&D) for 2 h at room temperature. Finally, peroxidase-conjugated rabbit anti-mouse or goat immunoglobulins (Dako) were added for 1 h at room temperature followed by extensive washing, before signals were developed by Plus or Advanced ECL chemiluminescence detection system (GE Healthcare) and a CCD camera (Fujifilm LAS-4000). 

### Dephosphorylation experiments

The specificity of the anti-pROR1 mAb and dephosphorylation of ROR1 in CLL cells was analysed using a lambda protein phosphatase enzyme (New England BioLabs, Hitchin, Hertfordshire, UK) which is a protein serine/threonine/tyrosine phosphatase. Briefly, 40 µg protein lysate from CLL cells without phosphatase inhibitor was incubated with 400 units of the phosphatase in the presence of protein metallo-phosphatases and MnCl_2_ at 37 °C overnight. Treated and untreated samples were analysed for ROR1 phosphorylation using western blot. 

### pROR1 intensity measurement

Phosphorylation intensity of ROR1 bands (105, 130 and 105/130 kDa) in CLL cells were analysed by western blot measuring the pROR1 intensity and the ROR1 (goat anti-ROR1 pAb) intensity by the ImagJ1.44p software (National Institute of Health, USA). The pROR1/ROR1 intensity ratio after deduction of the background intensity was calculated.

### Separation of nuclear and cytoplasmic proteins

Freshly prepared PBMCs of CLL patients were washed twice in PBS before lysates were prepared using NE-PER®Nuclear and cytoplasmic extraction reagents (Thermo Scientific), according to manufacture’s instructions. To confirm that proteins were localized to the correct fraction, western blot analysis was performed using anti α/β-tubulin (Cell Signaling Technology, Danvers, MA, USA), histone H3 and nucleolin antibodies (Abcam, Cambridge, UK) before analysing the expression pattern of ROR1 isoforms in the same preparation.

### Statistical analysis

Statistical analyses were performed using the chi-square test, independent sample T-test and Mann-Whitney U-test, using the Epi-Info (Version 6, November 1993) and SPSS statistical package (SPSS Inc., Chicago, IL, USA). The cut-off for significance was set to p < 0.05.

## Results

### ROR1 isoforms in CLL cells

**Figure 1:**
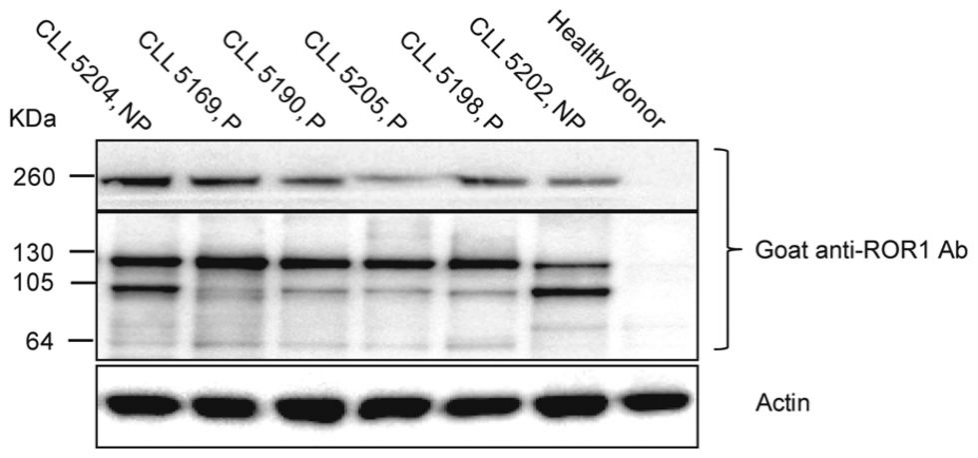
ROR1 isoforms in CLL. Representative experiments showing the presence of 260, 130 and 105 kDa bands in CLL patients. P indicates progressive disease and NP non-progressive disease. A healthy donor served as negative control.

Cell lysates from CLL samples were immunoprecipitated using a rat anti-ROR1 mAb. A polyclonal goat anti-human ROR1 Ab was used for immunoblotting. A band of 105 kDa was identified considered to represent the native unglycosylated ROR1 as well as a 130 kDa band supposed to represent the fully glycosylated monomer [5,6,9]. Representative blots are shown in Figures 1. Of these two bands, one or the other seemed to dominate in individual patients. A 260 kDa band was also noted, probably representing dimerized ROR1 (homo or heterodimerization), as the 260 kDa band was only noted under non-reducing conditions. This is expected as disulfide bridges will be reduced and dimers converted to monomers under reducing condition.

The expression of different ROR1 protein isoforms varied between patients. The unglycosylated ROR1 isoform (105 kDa) was more frequently seen in non-progressive (14 out of 17) as compared to progressive (10 out of 21) patients (p=0.03). 

### Specificity of the anti-pROR1 mAb

**Figure 2:**
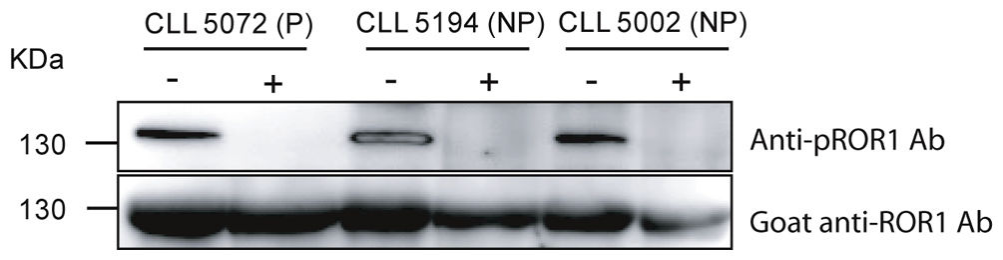
Specificity of anti-pROR1 antibody. Western blot analysis of CLL lysates from 3 CLL patients (5072, 5194, 5002) treated with a lambda phosphatase enzyme. **-**: enzyme-untreated sample, **+**: enzyme-treated sample. PVDF blot was stripped and reprobed with a goat anti-ROR1 polyclonal Ab.

A phosphatase enzyme removing all phosphate groups from phosphorylated proteins was used to treat CLL lysates. A representative experiment is shown in Figure 2. The anti-pROR1 mAb detected a 130 kDa ROR1 band in samples not treated with the enzyme, but a weak or no band in lysates treated with the enzyme. The same blot was also probed with a goat anti-ROR1 Ab. ROR1 band was detected in both untreated and enzyme treated samples (Figure 2A). Immunoprecipitated ROR1 (130 kDa) from CLL cells using a goat anti-human ROR1 Ab could be detected by the anti-pROR1 mAb (Figure 2B). The anti-pROR1 mAb was also tested in ELISA, using a recombinant extracellular (KNG) part of the ROR1 protein as well as in cell surface staining of CLL cells. The pROR1 mAb did not react with the extracellular ROR1 protein and did not stain the cell surface of CLL cells (data not shown). The results suggest that our anti-pROR1 mAb reacts with a phosphorylated intracellular part of the ROR1 molecule.

**Figure 3:**
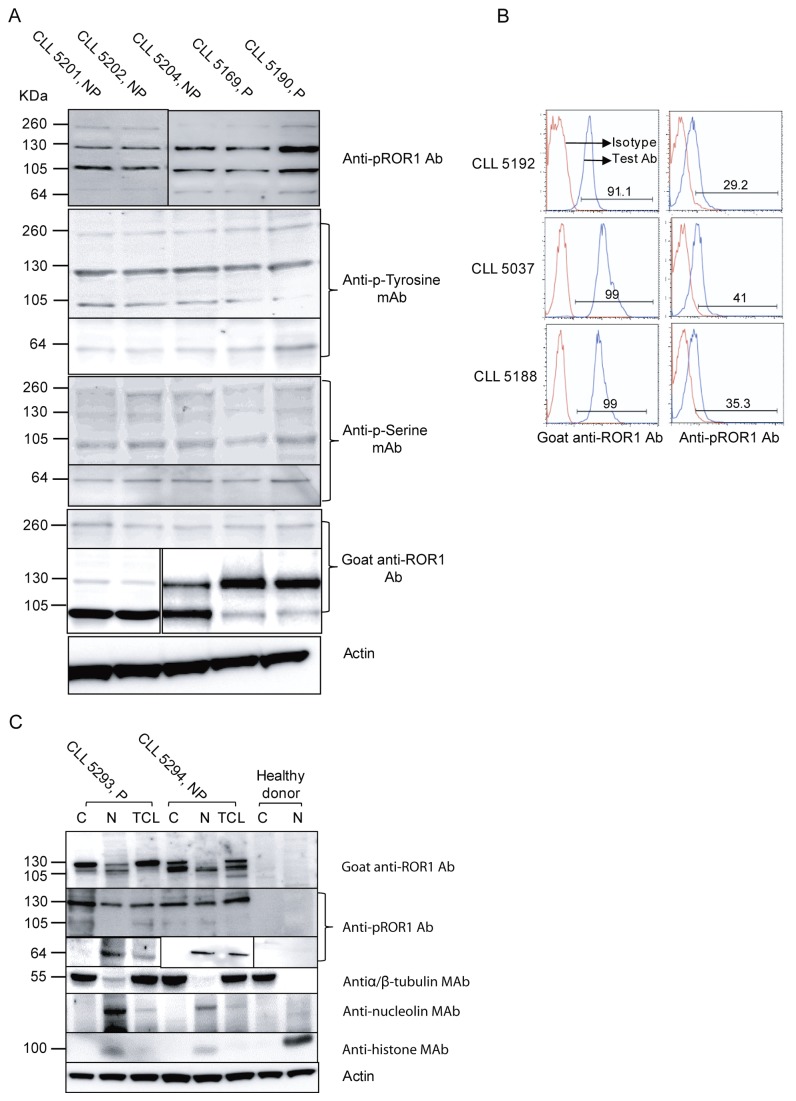
ROR1 isoforms and phosphorylation in CLL patients. Representative experiments using non-immunoprecipitated CLL cell lysates from 5 CLL patients showing phosphorylated dimerized ROR1 (260 kDa), fully glycosylated (130 kDa) and non-glycosylated (105 kDa) ROR1 molecules (A). NP indicates non-progressive disease and P progressive disease. As controls, CLL cell lysates immunoprecipitated with a non-relevant mAb (mouse IgG1 isotype) were used. No bands could be seen. Furthermore, in PBMC of healthy donors, no bands were detected (data not shown). Representative experiments of PBMC using surface staining for ROR1 (left panel) and intracytoplasmic staining for pROR1 (right panel) in three CLL patients (B). Expression of ROR1 isoforms in the cytoplasm (C), nucleus (N) and total cell lysate (TCL) of leukemic CLL cells (C). Confirmation of protein localization was done using antibodies against α/β –tubulin, histone H3 and nucleolin before analysing the expression pattern of ROR1.

This antibody also detected a 64 kDa band (Figure 3A), noted in 13/17 patients with non-progressive disease and 14/21 patients with progressive disease.

### Phosphorylation of ROR1 in CLL cells

Phosphorylation of ROR1 in CLL cells was analysed using cell lysates prepared from freshly isolated PBMC of 38 CLL patients. ROR1 was constitutively phosphorylated in all patients (Figure 3A). The 260, 130, 105 and 64 kDa isoforms were phosphorylated both at tyrosine and serine residues. The results could be confirmed by intracytoplasmic staining of CLL cells using the anti-pROR1 mAb (Figure 3B). Immunoprecipitation of CLL cell lysates using a non-relevant IgG mAb did not show any bands. PBMC and normal B-cell lysates from healthy donors using a rat anti-ROR1 mAb for IP and probing with the anti-pROR1 mAb did not either reveal any bands (data not shown). 

**Figure 4:**
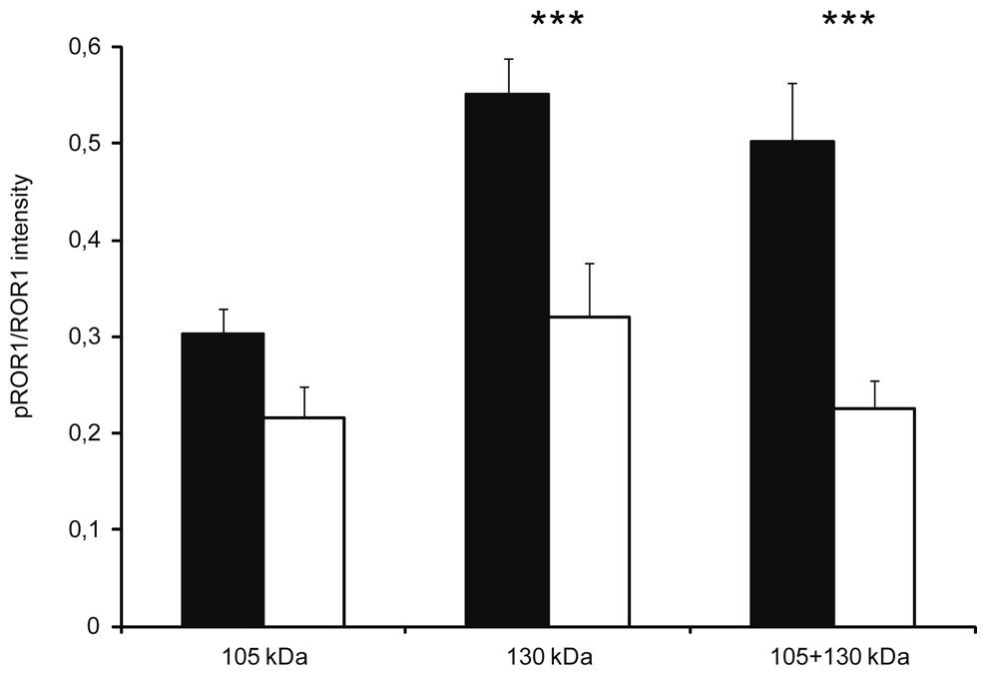
Differential phosphorylation of ROR1 isoforms in CLL in relation to disease activity. pROR1/ROR1 intensity (mean ± SEM) of the 105, 130 and combined 105+130 kDa bands in progressive (■) (n=21) and non-progressive (☐) CLL patients (n=17). *** p= 0.001.

The phosphorylation intensity of the 105 and 130 kDa bands in progressive and non-progressive CLL patients was also compared. There was no difference in the phosphorylation intensity of the 105 kDa band comparing patients with progressive and non-progressive disease at the time of testing. However, the degree of phosphorylation of the 130 kDa band was significantly higher in progressive than in non-progressive disease as was the case when measuring the intensity of both isoforms together (Figure 4). 

### Cellular localization of ROR1 isoforms

The localization of the ROR1 isoforms in the cytoplasm and nucleus respectively in leukemic cells was examined. Antibodies against α/β-tubulin (a cytoplasmic protein), nucleolin and histone H3 (nuclear proteins) were used to checked the purity of the nucleus and cytoplasmic fraction of the leukemic cells. Phosphorylated 105-130 kDa ROR1 isoforms were localized both to the cytoplasm and nucleus. The phosphorylated 64 kDa ROR1 isoform was only seen in the nucleus. Results of 2 CLL patients and one healthy control are shown in Figure 3C.

### Anti-ROR1 mAb induced dephosphorylation of ROR1

**Figure 5:**
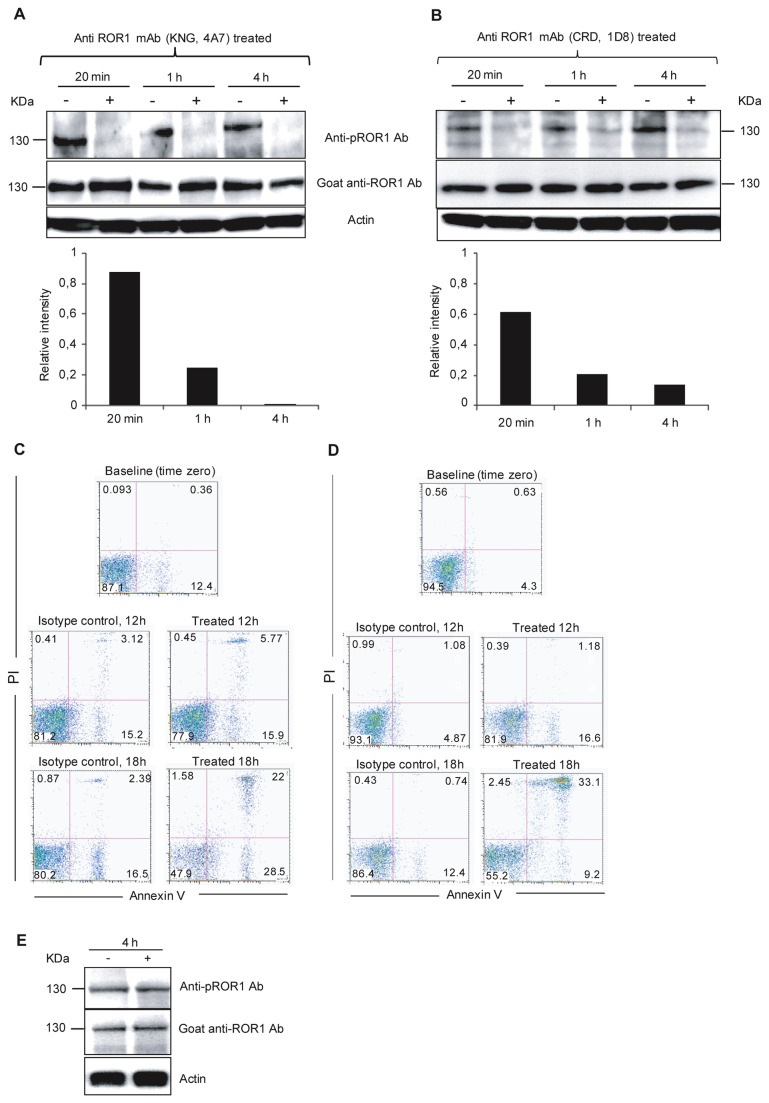
Anti-ROR1 mAbs induced dephosphorylation of ROR1 and subsequent apoptosis of CLL cells. Representative experiments of time-dependent ROR1 dephosphorylation and apoptosis in vitro incubated with an anti-KNG (4A7) (A and C) and anti-CRD (1D8) (B and D) anti-ROR1 mAb respectively. Representative experiments from two progressive (CLL 5102 and CLL 5116) CLL patients are shown. Leukemic cells were incubated for 20 min, 1, 4 hours with a non-relevant IgG1 isotype control mAb (-) and the ROR1 mAbs (+). Cells were harvested and lysed for western blot experiments (A and B). The non-relevant isotype control mAb did not induce dephosphorylation of ROR1 (130 kDa) while the anti-ROR1 mAb induced dephosphorylation already after 20 min, which increased by time. The intensity of pROR1 was measured by ImageJ software. The ratio of pROR1/ROR1 intensity of treated sample to pROR1/ROR1 intensity of untreated sample (relative intensity) is shown in a histogram. A value <1 indicates dephosphorylation of ROR1 after treatment with the anti-ROR1 mAb compared to the non-relevant isotype control mAb. Apoptosis of CLL cells (Annexin V^+^/PI^+^) after 0, 12 and 18 h incubation with the anti ROR1 mAbs without the addition of immune effector cells or complement (C and D). An anti-CD20 mAb (ofatumumab) did not induce dephosphorylation of ROR1 after 4h incubation in two CLL patients. A representative experiment is shown for one of the CLL patients (E).

We have previously shown that in vitro incubation of leukemic cells with anti-ROR1 mAbs alone induced apoptosis of CLL cells [8]. Here we examined ROR1 phosphorylation in apoptotic CLL cells induced by our self-produced anti-ROR1 mAbs (KNG 4A7 and CRD 1D8) [8]. CLL cells from 5 (two progressive and three non-progressive) patients were studied. Representative experiments are shown in Figure 5, one for the anti-KNG mAb (Figure 5A and C) and one for the anti-CRD mAb (Figure 5B and D). Cell lysates were probed with the anti-pROR1 Ab after 20 min, 1 h and 4 h of incubation with the ROR1 monoclonal antibodies. Apoptosis/necrosis (Annexin V^+^/PI^+^) was measured after 4 h, 12 h and 18 h of incubation. Three CLL samples were exposed to the anti-KNG mAb and two samples to the anti-CRD mAb. Dephosphorylation of the 130 kDa band was observed already after 20 min of incubation with the anti-ROR1 mAbs (Figure 5A and B). Furthermore, a weak dephosphorylation could also be noted for the 105 and 64 kDa bands respectively (data not shown). A non-relevant isotype control mAb (IgG1) and the anti-CD20 mAb (ofatumumab) did not induce dephosphorylation of ROR1 (Figure 5E). After 4 h of incubation with the anti-ROR1 mAbs, only few cells entered apoptosis/necrosis (5-4%). At 12 and 18 h, 22-18% and 52-45%, respectively of leukemic cells were apoptotic/necrotic (non-relevant IgG1 isotype control mAb: 19-7 % and 20-14%). The results suggest that dephosphorylation of ROR1 was specific for both the anti-KNG (4A7) and anti-CRD (1D8) mAbs and preceded apoptosis. 

## Discussion

Different isoforms of ROR1 (Table S1) could be shown to be present in CLL cells. The most prominent bands were the 105 and 130 kDa isoforms which were localized both to the cytoplasm and nucleus. We could also demonstrate the presence of dimerized ROR1 (260 kDa). The other member of the ROR family, ROR2 has been shown to be both homodimerized [17,18] and heterodimerized [19]. 

Post-translational modifications of ROR1 might be of functional significance. It was shown that ROR1 displayed a non-unique glycosylation pattern in CLL [9], similar to our findings. In ROR1, there are 7 potentially aspargine residues for N-linked glycosylation and more that 24 serine and threonine residues (http://www.cbs.dtu.dk/services) that might be O-glycosylated contributing to a varying glycosylation pattern. This may differ from patient to patient and between cells of an individual CLL clone. In this study, as well as in our previous report [6], we could show the presence of 100-130 kDa ROR1 glycoforms in CLL cells as has also been shown by Kaucka et al [9]. Kaucka and co-workers demonstrated that treatment of ROR1 transfected cells with tunicamycin (inhibiting N-linked glycosylation) and brefeldin induced deglycosylation of ROR1, yielding 100 and 115 kDa bands, while non-treated cells expressed a 130 kDa ROR1 band. Before treatment of CLL cells with N-glycosidase F, the 130 kDa ROR1 was the most prominent glycoform and deglycosylation inhibited surface expression of ROR1. Our present study confirms and extends those results, showing different ROR1 isoforms in CLL cells. The complete non-glycosylated (immature) ROR1 protein (105 kDa) was more frequently expressed in patients with non-progressive disease as compared to those with progressive disease, indicating that glycosylation may relate to disease activity as has been shown for other diseases [20]. 

A truncated ROR1 has been described in fetal CNS tissues, adult human CNS tumors, leukemia/lymphoma cell lines and in a variety of human cell lines of neuroectodermal origin using northern blot analyses [4]. The truncated ROR1 contained 388 amino acids without posttranslational modifications (approximately 40 kDa). This isoform was suggested to be derived from alternative splicing [4] or a cleaved C-terminal fragment (CTF) produced by surface proteolytic enzymes [21]. The size of this transcript (40 kDa) was different from what we found (64 kDa). 

The phosphorylated 64 kDa ROR1 isoform was only present in the nucleus. The cytoplasmic domain of ROR1 has been suggested to be a transcription factor but the target gene(s) are not known [22]. The nuclear localized cytoplasmic domain of ROR1 might play a role in cell migration and cytoskeleton remodeling, by upregulating genes involved in the regulation of the actin cytoskeleton, including radixin (RDX), ezrin (EZR), son of sevenless homolog 2 (SOS2) and caldesmon 1 (CALD1) genes [23]. A similar phenomenon has been described for other RTKs such as EGFR [24], ErbB2 [25], ErbB3 [26], ErbB4 [27] and VEGFR2 [28] where truncated versions had functional implications for the growth of the tumor cells. In breast cancer patients, a C-terminal fragment of HER2 (p95 HER2) lacking the extracellular part has been shown to correlate with a poor prognosis and resistance of HER2 positive cells to trastuzumab therapy [29]. 

We could also demonstrate that the ROR1 isoforms were constitutively phosphorylated at serine and tyrosine residues. Covalent binding of phosphate groups to serine, threonine and tyrosine residues are important for regulating protein activities [30]. Constitutive phosphorylation including ligand-dependent receptor dimerization/oligomerization of RTKs or ligand-independent is of importance for cell signaling [31,32]. ErbB2 or HER2/neu, as well as ROR1 are members of the type I RTKs contributing to the malignant phenotype of several human cancers. High expression of HER1/2, VEGFR_2_/KRD as well as estrogen receptors and their tyrosine phosphorylation in breast cancer correlated to a poor prognosis [31,33-35]. Our finding that phosphorylation of the mature ROR1 isoform (130 kDa) was significantly higher in progressive than in non-progressive CLL patients is of interest. Furthermore, we have also shown a significantly higher frequency of surface ROR1 expressing leukemic cells in progressive as compared to non-progressive CLL patients [36]. Mature ROR1 might also to be preferentially surface expressed [9]. Thus, there seems to be a relation between ROR1 maturity, phosphorylation and disease activity in CLL. Interestingly, induction of ROR1 expression in TCL1 transgenic mice accelerated the development of CLL [37].

It was recently shown that mouse ROR1 was phosphorylated at serine position 652 [30] located in the activation segment of ROR1, both in the human and mouse. This serine residue may be an important site to be triggered by serine/threonine kinases. The NetPhosK bioinformatic software (http://www.cbs.dtu.dk/services/) for prediction of kinase specific phosphorylation sites revealed that two tyrosine residues at positions 641 and 646 of the intracytoplasmic part of ROR1 might have a high probability to be phosphorylated by tyrosine kinases. There are three tyrosine residues which might be potentially phosphorylated located at positions 186, 828 and 836 respectively. The peptide used for production of the anti-phospho-ROR1 Ab was within the activation segment of the two tyrosine and one serine residues. 

It is not clear if constitutive phosphorylation of the ROR1 molecule at tyrosine [38-40] or serine [41,42] residues are due to autophosphorylation or not. Constitutive tyrosine phosphorylation triggered by other tyrosine kinases is a general phenomenon observed for RTKs [43]. It was recently shown that ROR1 had a low degree of autophosphorylation (low catalytic activity), the same as for ErbB3, but not as strong as for ErbB2 [44]. The Met oncogene has been suggested to phosphorylate ROR1 at tyrosine residues as a consequence of transphosphorylation [44]. The Met oncogene has also been shown to be overexpressed in CLL cells but not in normal B cells [45]. However, the Met oncogene is not the only kinase that might phosphorylate ROR1, as a high level of ROR1 phosphorylation was observed in cell lines with normal Met expression and activity [44]. Further studies are needed to understand the mechanisms of ROR1 phosphorylation which seems to be of importance for the malignant phenotype of many tumor cells [44]. However, no mutational activations have been shown for the ROR1 gene [6].

Binding of a ROR1 specific mAb to the external parts of the receptor, both the KNG and CRD domains, was associated with a rapid dephosphorylation of ROR1 preceding apoptosis of CLL cells. Apoptosis occurred in the absence of immune effector cells or complement. If ROR1 phosphorylation is ligand independent, binding of a mAb to the extracellular part may induce conformational changes and phospho-tyrosine residues at the C-terminal may be triggered by phosphatases to dephosphorylate ROR1, affecting ROR1 associated downstream signaling pathways [38-40,42]. ROR1 phosphorylation might also be ligand dependent and binding of the mAb may prevent binding of the ligand. Wnt5a has been suggested to be a ligand of ROR1 [46] and CLL cells have been shown to produce Wnt5a [47]. Further studies are needed to clarify the mechanism of action of our anti-ROR1 specific mAbs.

In summary, different ROR1 isoforms could be detected in CLL cells, which were constitutively phosphorylated. Phosphorylation was more pronounced in patients with progressive than non-progressive disease. Anti-ROR1 mAbs induced dephosphorylation of ROR1 in CLL cells preceding apoptosis of the CLL cells. Further studies on the biology of ROR1 in CLL and other malignancies are warranted to develop ROR1 targeting therapeutics.

## Supporting Information

Table S1 **ROR1 isoforms.**
(DOC)
