# Supplementary material for: The Tyrosine Kinase Receptor ROR1 Is Constitutively Phosphorylated in Chronic Lymphocytic Leukemia (CLL) Cells
Source: PLoS One. 2013 Oct 24;8(10):e78339. doi: 10.1371/journal.pone.0078339 (PMC3813472; doi:10.1371/journal.pone.0078339)
Supplement: Table S1 — ROR1 isoforms. (DOC) [file pone.0078339.s001.doc]

**Table.S1:** ROR1 isoforms

| **Molecular weight (kDa)** | **Length (aa)** | **Membrane bound** | **Glycosylation status** | **Tyrosine/serine**  **phosphorylation** | **Nuclear**  **localization** | **Reference** |
| --- | --- | --- | --- | --- | --- | --- |
| 130 | 937 | + | + | + | + | Present study  6, 9, 22 |
| 115 | 937 | + | + | + | NI | Present study  9 |
| 105 | 937 | NI | - | + | + | Present study  6, 9  ROR1-001, ENSP00000360120 |
| 64 | NI | NI | NI | + | + | Present study |
| 50 | NI | NI | NI | NI | NI | 5 |
| 40 | 393,  388 | NI | NI | NI | NI | 4  ROR1-002,  ENSP00000360121, ROR1-201, ENSP00000441637 |

NI = no information
